# Understanding the Impact of Migration on the Work Ability of Nurses: A Cross-Sectional Comparison Between Germany and Kosovo

**DOI:** 10.3390/nursrep15060203

**Published:** 2025-06-04

**Authors:** Petrit Beqiri, Flaka Siqeca, Rona Karahoda, Vjose Hajrullahu, Olga Agahi, Naime Brajshori

**Affiliations:** 1 Department of Nursing, Faculty of Health and Nursing Sciences, Heimerer College, Veranda D4, Kalabri Neighborhood, 10000 Pristina, Kosovo; petrit.beqiri@kolegji-heimerer.eu (P.B.); flaka.siqeca@kolegji-heimerer.eu (F.S.); 2 Institute for Health and Nursing Science, Faculty of Medicine, Martin Luther University Halle-Wittenberg, 06112 Halle (Saale), Germany; 3 Department of Public Health (DPH), University of Basel, Medizinische Fakultät, Bernoullistrasse 28, CH-4056 Basel, Switzerland; 4 Research Unit, Heimerer College, Veranda D4, Kalabri Neighborhood, 10000 Pristina, Kosovo; rona.karahoda@kolegji-heimerer.eu; 5 Department of Management of Healthcare and Health Institutions, Faculty of Health and Nursing Sciences, Heimerer College, Veranda D4, Kalabri Neighborhood, 10000 Pristina, Kosovo; vjosa.hajrullahu@kolegji-heimerer.eu; 6 Institute of Southeast Europe for Health and Social Policy, Veranda D4, Kalabri Neighborhood, 10000 Pristina, Kosovo; olga.agahi@isee-hsp.net

**Keywords:** nurses, migration, work ability, cross-sectional

## Abstract

**Background/Objectives:** Comparative analysis of work ability factors between German nurses without a migration background and Kosovar nurses working in Germany as nurses with a migration background. **Methods**: This is an observational cross-sectional study. We surveyed 814 nurses from hospitals in Kosovo and Germany using self-administered questionnaires. We used the Nurses Working Capability (WiN) Screening Manual to analyze work ability factors. Data analysis involved descriptive statistics, ANCOVA, and Bonferroni multiple comparison tests. **Results**: Of the 40 target factors, 17 showed significant differences. There were notable distinctions between German and Kosovar nurses in Germany. Concerning health and personal resources, Kosovar nurses in Germany reported poorer physical (M = 3.71) and social health (M = 3.83) but better mental health (M = 3.53) and fewer cognitive stress symptoms (M = 3.60) than German nurses (M = 3.97, M = 4.05, M = 3.40, and M = 4.00, respectively). As to the work-related factors, Kosovar nurses in Germany faced higher emotional demands (M = 3.33), felt greater time pressure (M = 3.43), and had a more negative view of the organizational structure (M = 2.09) but rated supervisor feedback more positively (M = 3.24) and faced fewer patient-related stressors (M = 2.16) relative to German nurses (M = 2.64, M = 2.64, M = 2.82, M = 2.76, and M = 3.09, respectively). **Conclusions**: Kosovar immigrant nurses in Germany face distinct challenges related to physical and social health, higher emotional demands, and time constraints; they also have a more negative view of organizational structure than native German nurses. The possible causes of these differences may be attributed to migration-related stress, cultural and environmental adaptations, varying job expectations, or organizational experiences.

## 1. Introduction

The migrant workforce is becoming essential for meeting the need for qualified nursing personnel in various countries. Understanding how their experiences and challenges affect their ability to work is vital to ensuring that their workplaces provide good working conditions, so that they feel supported, both professionally and in building a new life abroad. Nurses’ working conditions need to be studied, improved, and protected to ensure these essential workers can sustain their ability to work for as long as they need.

### 1.1. Global Context

The United Nations demographic projections suggest that the global population could reach around 8.5 billion by 2030, 9.7 billion by 2050, and 10.4 billion by 2100 [1]. This global demographic shift means that the current nursing workforce will need to provide high-quality care to an increasing number of individuals who are not only growing older but also have complex needs [2]. On the other hand, and especially following the COVID-19 pandemic, nurses face an increased workload, work-related stress and burnout, continuous demands for learning new skills and higher productivity at work, and the necessity to be flexible regarding time and location [2].

### 1.2. Nursing Shortage

At the same time, many healthcare systems face nursing shortages. Before the COVID-19 pandemic, the global shortage of nurses was estimated at 5.9 million [3]. However, the global projections of the International Council of Nurses suggest that over 13 million nurses will be required to match the demand by 2030 [4]. The European Regional Office of the World Health Organization highlights this challenge as a key factor blighting the nursing workforce across European countries [5]. An important influence mentioned by policymakers contributing to the mismatch between supply and demand for the nursing workforce is migration, which has been a growing concern for many countries in Europe and beyond, further burdening an already strained supply of nursing staff [6].

According to estimates, Germany will be facing a nursing shortage of 500,000 in 10 years [7]. The country has a very high percentage of foreign nurses—16%, which is instrumental in closing the nursing shortage [8]. A survey conducted among healthcare workers in Kosovo indicated that 14.43% aspired to migrate, with 23.68% reporting an increased chance of migrating after the pandemic [9].

### 1.3. Work Ability Factors

The concept of work ability (WA) became a topic of interest for Finnish researchers in 1980 when Ilmarinen and colleagues from the Finnish Institute of Occupational Health developed the Work Ability Index (WAI) for better understanding of how long individuals should expect to continue working while facing the workforce challenges of the time [10]. The ability to work is determined by the employee’s resources and by the work itself. The former includes physical, mental, and social skills, health, competence, and individual values [11], while the latter is determined by the work content, organization, social environment, and management [11].

The WAI index, though widely used since its development in many fields, was not deemed suitable for use in nursing. Hence, in 2010, German researchers developed a screening manual based on the Work Ability Index (WAI) covering all areas of work ability, such as health, training/competence, values/attitudes, and work, which can be used specifically for nursing. It is referred to as the Work Ability in Nursing (WiN) Screening Manual [12].

### 1.4. Migrant Workforce in Germany

Germany is one of the primary high-income countries attracting skilled workers, such as nurses [13], making it an ideal context for studying factors related to work ability among nurses from migrant backgrounds. Since 2022, the growth in the nursing population has been attributed solely to nurses from abroad. The percentage of foreign nurses in Germany has risen steadily in recent years. According to the Federal Employment Agency [9], 16% of nurses in Germany now come from other countries, compared to just 5% in 2013. In 2018, nearly half of foreign nurses came from various European countries, while in 2023 this percentage fell to 35%, with the largest proportion—65%—of foreign nurses coming from countries outside the EU [9].

### 1.5. Challenges Faced by Migrant Nurses

Some challenges experienced by migrant nurses include communication issues and language barriers, which may persist for several years after moving to the host country [14]. Their social and mental health often suffers, and their feelings of social isolation increase [15]. Migrant nurses also face issues around the recognition of their qualifications and often work below them [16,17], while also meeting with racism and discrimination, often the worst aspects of the migrant experience [18]. Among other challenges are the attitudes of senior staff, a perceived lack of support, and the feeling of being held back from accessing opportunities to progress professionally due to a lack of recognition of qualifications. Social support, or a lack thereof, is another aspect worth considering. Whether nurses come to the new country alone, with their families, or with a group of other nurses is a factor in the quality of their experience, as many face homesickness and difficulties with leaving their families [19].

Understanding the experiences and challenges of migrant nurses can aid policymakers in developing policies that adequately respond to pressing workplace issues and ensure good working conditions for all, which is paramount to tackling the challenges associated with the demographic and workforce shifts of the future.

### 1.6. Research Problem and Significance of the Study

In this connection, some critical issues emerge that the present study aims to address. Firstly, the number of third-country nationals (individuals from non-EU countries) residing in Germany is increasing—a trend also evident among nurses. Recent data show that 12.6–16% of nurses in Germany have a migration background [9,20]. Secondly, a literature search covering studies from December 1996 to July 2017 did not find direct studies on the assessment, maintenance, or promotion of work ability among this demographic. As such, this study is among the first to explore this crucial gap. By collecting and analyzing comparative data from different groups, the aim is to identify potential differences that could inform the development of integrated programs for damage prevention and health promotion.

### 1.7. Study Objectives

More specifically, the objective of the present study is to investigate the differences in work ability among migrant and non-migrant nurses in Germany using the WiN Screening Manual with a focus on implications for workforce planning. We aim to understand how migration factors affect nurses’ workability by comparing the workability factors among German and Kosovar nurses without a migration background, working in their respective countries, to German and Kosovar nurses working in Germany, to examine the experience of nurses without a migration background as compared to those with this background.

### 1.8. Hypothesis

**H₀1 (Null Hypothesis):**  *There is no significant difference in work ability between nurses with and without a migration background in Germany.*

**H1.**  *There is a statistically significant difference in work ability between nurses with and without a migration background in Germany.*

**H₀2 (Null Hypothesis):**  *Migration-related stressors do not influence the work ability of nurses with a migration background in Germany.*

**H2.**  *Migration-related stressors influence the work ability of nurses with a migration background in Germany.*

## 2. Materials and Methods

### 2.1. Design and Setting

This observational cross-sectional study was conducted in 2017 in German and Kosovar tertiary and secondary hospitals. To examine the influence of contextual factors such as culture and history on the Work Ability Index, the study focused on nurses with a migrant background. The WAI version used for the study was designed specifically for nurses, and migrant nurses were studied to isolate the cultural and historical variables. Kosovo was chosen as a case study, as it is relevant to the German healthcare system, with a significant number of Kosovars working in Germany. Kosovo was selected deliberately, as it is a small and relatively culturally homogeneous country. This homogeneity allows for minimizing confounding variables and better isolating the effects of cultural and historical background on work ability. Choosing a larger and more diverse country, such as India or China, for example, would introduce substantial variability, thus complicating the analysis.

The facilities in Germany were secondary and tertiary care hospitals. The specialties included in the survey were as follows: Dermatology, Hemodialysis, Physiotherapy, Obstetrics, Infectious Diseases, Intensive Care, Internal Medicine, Surgery, Neurology, Orthopedics, Pediatrics, and Psychiatry. All employees from the medical service, nursing staff, medical–technical service, and functional and technical service who were employed at the hospital were surveyed. All other employees were excluded from the survey.

To compare with similar facilities in Germany, the University Hospital in Prishtina and two regional secondary care hospitals in Mitrovica and Vushtrri, which offer comparable specialties, were selected in Kosovo. However, in Kosovo, unlike Germany, there is not as much differentiation among the nursing skill levels. All nurses working in the profession are required to be registered and have a diploma.

### 2.2. Sample and Procedure

This study used self-administered questionnaires and included a sample of 814 nurses divided into three groups: (1) 503 German nurses in German hospitals (825 distributed, response rate = 72.7%); (2) 251 Kosovar nurses in Kosovo hospitals (300 distributed, response rate = 83.6%); and (3) 60 Kosovar nurses in German hospitals. German nurses completed the questionnaire in German, Kosovar nurses completed it in Kosovo in Albanian, and Kosovar nurses in Germany could choose either Albanian or German.

Participants from the first and second sample groups were selected from nurse registries at secondary and tertiary care hospitals in Kosovo and Germany. Due to challenges in reaching the final group using the original sampling method, participants for the last sample were recruited through snowball sampling.

Participants received questionnaires at the end of their shifts or during team meetings. Participation was voluntary, and all responses were submitted anonymously within eight weeks of distribution. As per data protection regulations, all collected data were handled with strict confidentiality and processed exclusively for scientific research purposes.

### 2.3. Variables and Measurements

Demographic information was collected on age (in years), gender, and years of work experience.

To assess workability, we used the Nurses Working Capability (WiN) Screening Manual, developed in German by Girbig et al. [12]. This tool integrates items from four validated core instruments: the Work Ability Index (WAI) [10]; the Copenhagen Psychosocial Questionnaire (COPSOQ)—Short German Version [21,22]; the Diagnosis of Health-Promoting Work analysis tool (DigA) [23]; and the Activity and Work Analysis Procedures (TAA) [24]. In addition, selected items were drawn from the Copenhagen Burnout Inventory (CBI) [25], the EQ-5D-5L [26], and the Work–Family Conflict Scale [27] to ensure comprehensive coverage of all relevant areas.

The instrument was created using German. The original German version was used for German nurses. It was translated into Albanian for the Kosovars. This translation followed a specific 10-step procedure for translation of validated instruments found in ISPOR [28]. The instrument was first translated from German to Albanian, then from Albanian to German by a different translator.

The WiN Screening Manual consists of 185 items, grouped into the following domains: Health (39 items), Training/Competence (15 items), Values/Attitude (8 items), and Work (123 items). All items were measured on a five-point Likert scale. For this study, these items were grouped into 40 key target factors of work ability, consistent with the instrument. These factors were derived through theoretical alignment with the work ability framework and represent a combination of individual resources and work-related demands relevant to nursing performance.

A full list of the 40 target factors, their source instruments, and item counts can be found in Appendix A.

### 2.4. Ethical Considerations

Before data collection began, ethical approval was obtained from the University Clinical and Hospital Service of Kosovo (SHSKUK). Additionally, the Faculty of Medicine of Martin Luther University Halle granted ethical permission. Participants’ confidentiality and anonymity were ensured, and all relevant information was clearly explained. Each participant had the right to withdraw from the study at any time.

### 2.5. Data Analysis

Descriptive statistics were computed for the demographic variables. Continuous variables were summarized using central tendency (mean) and spread (range) measures, while categorical data were expressed as percentages. The mean value, standard deviation, and standard error were calculated for each Likert-scale item.

To assess differences between the three groups, we conducted a covariance analysis (ANCOVA), with the significance level set at *p* < 0.05. For significant work ability factors, we followed up with a multiple comparison test using the Bonferroni method to ensure that the general significance level remained consistent in all pair-wise group comparisons.

Data analysis was conducted using SPSS (version 21) [29].

## 3. Results

### 3.1. General Demographic Characteristics

Most Kosovar nurses working in Kosovo are female (80.5%), while the opposite trend can be observed in the other two groups, where most nurses are male. In general, nurses in Kosovo were older than German and Kosovar nurses working in Germany. This age difference is also reflected in their work experience: Kosovar nurses in Kosovo typically have 17 years of experience, compared to 9 years for German nurses in Germany and 10 years for Kosovar nurses in Germany (Table 1).

### 3.2. Work Ability Factors Among Nurses

Of the original 40 target factors, differences between the groups were found for 17 target factors. Table 2 shows the mean scores, standard deviations (SD), and standard errors (SE) for these 17 different target work ability factors across three participant groups: Kosovar nurses working in Kosovo, German nurses in Germany, and Kosovar nurses in Germany.

#### 3.2.1. Health Domain

In the Health domain, Kosovar nurses in Kosovo scored highest on all three health factors—physical (M = 4.11), mental (M = 3.79), and social (M = 4.11)—suggesting that their self-perception is quite high. While German nurses fell in the middle for physical (M = 3.97) and social health (M = 4.05), Kosovar nurses in Germany scored lowest of all on these two—physical (M = 3.71) and social health (M = 3.83). However, the difference in social health was not statistically significant. This could be due to the added strain of migration, change in diet, physical exercise, and a reduced social network compared to the one available at home. It is also possible that health perception varies and is rated differently across cultures; the job demands, and varying levels of cultural and social support could all be factors. Notably, mental health was rated higher by Kosovar nurses in Germany (M = 3.53) than by their German colleagues (M = 3.40), although the difference was not statistically significant. The cognitive stress symptoms were highest in Kosovar nurses in Kosovo (M = 4.01), which could potentially be due to stressful working conditions in Kosovo, followed by German nurses (M = 4.00), with the lowest score for Kosovar nurses in Germany (M = 3.60). This could reflect better working conditions for Kosovar nurses in Germany, but also indicates a difference in the perception of cognitive stress by both groups working in Germany.

#### 3.2.2. Values Domain

In the Values domain, the total motivation to work (M = 1.93) and total general motivation (M = 1.95) were rated rather low across all three participant groups. German nurses scored highest among the three groups for both (M = 2.10 and M = 2.09, respectively). Kosovar nurses in Germany rated emotional demands highest (M = 3.33), while their German counterparts rated it the lowest (M = 2.64) of all groups. This suggests that migrant workers may feel an additional emotional load. Patient-related stressors, however, were rated higher by German nurses (M = 3.09) than by their Kosovar counterparts in Germany (M = 2.16)

#### 3.2.3. Work Domain

Within the Work domain, the “thoughts about the job” factor was rated relatively low overall (M = 1.54) across all three groups. However, German nurses scored unusually low for this factor (M = 0.11), which stands out compared to Kosovar nurses in Kosovo (M = 1.65) and Kosovar nurses in Germany (M = 1.73).

In the Demands sub-domain of the Work domain, Kosovar nurses in Germany reported higher scores on time pressure (M = 3.43) and work–family privacy conflict (M = 2.82) compared to the other groups. They also rated the leadership quality (M = 2.87), supervisor feedback (M = 3.24), and general job satisfaction (M = 2.39) higher than their colleagues in Kosovo, suggesting a more positive perception of workplace support in the host country. However, they scored lower on job satisfaction than their German colleagues (M = 2.76). General assessment of the organization was also rated the lowest among Kosovars in Germany (M = 2.09), but highest for their counterparts in Kosovo (M = 2.87).

### 3.3. Differences in Work Ability Factors Among Migrant and Non-Migrant Nurses

Following ANCOVA testing, nine target work ability factors differed significantly across participant groups. For these, Bonferroni-adjusted multiple comparison tests were conducted to identify specific group-level differences (see Table 3).

#### 3.3.1. Health Domain

In terms of physical health, significant differences were observed among the three groups. Kosovar nurses in Germany reported the lowest physical health scores, which might indicate a potential decline in physical well-being following migration. Better monitoring of occupational health and targeted support programs could be useful in tackling this issue.

No significant differences were found between German and Kosovar nurses working in Germany in the domain of mental and social health, suggesting a comparable experience in these areas. However, the cognitive stress symptoms among German nurses were significantly higher compared to the Kosovar group in Germany.

#### 3.3.2. Work Domain

Kosovar nurses in Germany reported significantly higher emotional demands than their German colleagues. This may be due to language barriers, the stress of adapting to a new culture, and the emotional labor in interactions with patients from different cultures. In contrast, they experience significantly lower patient-related stressors than their German colleagues and the Kosovar nurses in Kosovo. This could suggest better support from their institutions or fewer complex patient interactions, but could also potentially indicate underreporting due to adaptation or communication barriers.

Regarding time pressure, German nurses experienced significantly lower time pressure than their Kosovar colleagues in Kosovo and Germany. This may be due to better staffing ratios and workflow systems in Germany, which highlights the value of operational efficiency in reducing staff burnout.

Lastly, with regard to the general assessment of the organization, Kosovar nurses in Germany rated this factor lower than both German nurses and Kosovar nurses in Kosovo. This result could indicate potential dissatisfaction with the organizational culture, integration process, or management styles encountered in host institutions. In contrast, Kosovar nurses in Germany rated the supervisor feedback higher than both other groups.

## 4. Discussion

### 4.1. General Discussion

This study aimed to compare work ability factors between German nurses without a migration background and Kosovar nurses working in Germany, representing nurses with a migration background. As previously outlined, work ability is shaped by both personal resources and the work environment, and is defined as the combination of factors that enable an employee to perform work tasks effectively [11]. Our findings revealed several notable differences between Kosovar and German nurses working in Germany across both domains.

In terms of individual resources, represented by the health domain, Kosovar nurses in Germany rated their physical health and cognitive stress symptoms significantly lower than their German colleagues. Although the difference was not statistically significant, their social health was also rated lower, while the opposite trend was observed for mental health.

The lower physical and social health among migrant Kosovar nurses in Germany, compared to their counterparts in Kosovo, could be attributed to the significant disruption caused by migration. This includes a complete change in the environment and daily routines, diet, exercise routines, and overall lifestyle in the new environment. A systematic review from 2017 found that adapting to a new country’s behaviors, lifestyle, and language can make migrant nurses vulnerable to stressful experiences, which, in turn, may negatively affect their health. In addition, experiences of discrimination and bullying have also been shown to adversely affect the social well-being of migrant nurses, which aligns with our findings [30].

However, this study found that the migrant Kosovar nurses in Germany have better mental health and fewer cognitive stress syndromes than German nurses, which contrasts with the findings from several other countries [31,32,33]. This difference may be due to the nature of employment held by the participants in our study. In Germany, nursing is considered a highly skilled and respected profession, as opposed to the menial, precarious, and high-risk jobs often held by migrants in other studies.

When examining work-related factors affecting work ability, Kosovar nurses in Germany experienced higher emotional demands but lower patient-related stressors than their native German colleagues. The difference in emotional demands could stem from caring for patients from very different social and cultural norms compared to their own. Moreover, being away from loved ones and lacking a strong local support system may further intensify the feelings of high emotional demands among migrant nurses, in addition to the inherent nature of the nurses’ job, where dealing with the loss and suffering of others is common [30,34]. We also observed that German nurses reported significantly lower time pressure than their Kosovar migrant colleagues. This difference could be partly due to the strong cultural emphasis on punctuality and efficiency in the German workplace, to which migrant nurses must quickly adapt, potentially adding to their workload and perceived time-related stress.

Lastly, Kosovar nurses in Germany gave the lowest general assessment of the organization. This could indicate the discrepancy between the organizational structures at home and in the new country, potential integration issues, or differences in management styles leading to dissatisfaction. However, the same group showed the most positive assessment of supervisor feedback. This may reflect more structured feedback mechanisms in the host country or a greater appreciation for communication for migrant nurses who had to adapt to a new workplace culture.

### 4.2. Practical Implications for Policymaking

Our findings collectively highlight the need for focused support programs for migrant nurses and have important implications for healthcare policy and practice. Introducing orientation programs, language training, mental health services, and social integration initiatives may enhance the social and physical health of migrant nurses. Furthermore, training in cultural competence can assist in bridging gaps in emotional demands and stressors related to patients for both native and immigrant nurses.

More priority should be given to workforce retention strategies and tailored support for migrant nurses. Recruitment practices should focus not only on addressing shortages but also on nurses’ professional aspirations. Considering their needs for professional fulfillment, as well as organizational settings, conditions, attitudes, and the level of support from superiors, influences the level of commitment. Improvements in these areas would lead to more effective and better retention. Ensuring that nurses have a community they can rely on is also crucial to their adaptation [19]. The differences in emotional demands, potentially due to treating patients from very different social and cultural norms compared to those at home, could be evened out with appropriate cultural competence training. 

The findings also highlight disparities in physical and social health, with worse outcomes for migrant nurses; therefore, interventions focused on mental health support and work–life balance would be appropriate.

### 4.3. Limitations

This study has several limitations that we would like to acknowledge.

First, the sample size of Kosovar nurses in Germany was considerably lower than the other two groups. This may affect the generalizability of the findings and should be interpreted cautiously. However, we believe that the comparison between the core groups of German and Kosovar nurses in Germany still offers valuable insights.

Another important aspect to consider, though not a direct limitation of the study, is the difference in skill-grade structures among nurses between Germany and Kosovo. Germany has a wider range of educational backgrounds for this profession; in Kosovo, a tertiary degree is still required to practice as a nurse. These differences in training and experience might have also influenced some of the results of our study and should be considered when interpreting them.

A further limitation is the study’s cross-sectional design, which captures data at a single point in time. Although we collected a good number of work ability factors, most are too complex to be captured in a snapshot in a cross-sectional design and are also likely to change and evolve. A longitudinal design would allow for deeper insights into how these factors develop over time and might help establish causal relationships.

Given these limitations, the findings should be interpreted with caution, particularly when considering their generalizability beyond the specific study settings. Nonetheless, the results offer valuable insights into the work experiences of migrant nurses in Germany.

### 4.4. Recommendations

Given the practical implications outlined above, the following recommendations could guide policymakers in addressing the challenges identified in this study:

(a) Informing organizational leaders and raising awareness about the importance of incorporating specific migration-related factors into their Corporate Health Management (CHM) during the early planning and design stage (conceptual phase);
(b) Adapting and expanding assessment tools to capture specific migration factors;
(c) Training of experts and middle management responsible for implementing CHM measures in the use and application of the revised assessment tools;
(d) Adapting and expanding health circles and measures for hazard prevention and health promotion to include specific migration factors;
(e) Implementing measures that take migration factors into account;
(f) Conducting migration-sensitive evaluation.

In addition to work ability and health-promoting effects, adequately addressing migration-specific stressors within the CHM concept would lead to a sense of equal treatment and recognition among migrant employees. This could make it easier for them to identify with the organization. Ultimately, the CHM concept, expanded to include specific migration factors, could take a further step toward including a rapidly growing employee group worldwide and thus make a significant contribution to sustainable work ability promotion [20].

### 4.5. Future Research

Future research should focus on longitudinal studies to measure outcomes for migrant nurses over longer periods and measure the efficacy of interventions. Adding comparative analyses across various nations and professions, as well as incorporating qualitative data on nurses’ personal experiences, could help provide a more comprehensive understanding of the migrant workforce experience. Moreover, studies investigating the significance of organizational elements, such as work culture and management procedures, could offer a more thorough comprehension of the elements affecting work ability.

## 5. Conclusions

Migrant nurses face unique challenges that go beyond their professional duties. In addition to the demands of their work, they must navigate the challenges associated with moving to a new country, separation from family and friends, and coping with cultural and language barriers, which all hinder their integration into their respective host countries [35]. Their daily routines are disrupted, leading to poorer health outcomes. Lower social support can increase feelings of isolation and stress. Adapting to a new workplace culture, with different organizational styles and systems in place, can also pose difficulty.

To improve outcomes for migrant nurses, healthcare leadership should be made aware of the migration-related challenges these workers face. Such awareness could help inform their work plans and implementation strategies, which would help better support the integration of migrant nurses.

Moreover, conducting longitudinal research to assess the effectiveness of interventions for migrant nurses could provide valuable insights into the issues they face, as well as provide evidence on how best to support them. Understanding the factors that affect their work ability and addressing associated challenges through supportive policies and practices is crucial for maintaining a healthy and effective workforce.

## Figures and Tables

**Table 1:** Demographic characteristics of participants.

	N = 814	M	F	Mean Age (Range)	Work Experience (Range)
Kosovar nurses in Kosovo	251	19.5%	80.5%	40 (18–62)	17 (1–43)
German nurses in Germany	503	78.6%	21.4%	30 (21–63)	9 (1–42)
Kosovar nurses in Germany	60	77.7%	20.0%	35 (21–49)	10 (1–31)

**Table 2:** Descriptive statistics of target work ability factors.

Target Work Ability Factors	Participant Group	Mean	SD	SE
Physical Health	Kosovar nurses in Kosovo	4.11	0.04	0.69
	Kosovar nurses in Germany	3.71	0.17	1.26
	German nurses in Germany	3.97	0.03	0.57
	Total	4.00	0.68	0.02
Mental Health	Kosovar nurses in Kosovo	3.79	0.06	0.96
	Kosovar nurses in Germany	3.53	0.11	0.84
	German nurses in Germany	3.40	0.04	0.99
	Total	3.54	0.99	0.03
Social Health	Kosovar nurses in Kosovo	4.11	0.05	0.79
	Kosovar nurses in Germany	3.83	0.09	0.69
	German nurses in Germany	4.05	0.03	0.68
	Total	4.05	0.72	0.02
Cognitive stress symptoms	Kosovar nurses in Kosovo	4.01	0.05	0.84
	Kosovar nurses in Germany	3.60	0.10	0.73
	German nurses in Germany	4.00	0.04	0.80
	Total	3.98	0.81	0.03
Motivation to work	Kosovar nurses in Kosovo	1.71	0.04	0.63
	Kosovar nurses in Germany	1.45	0.67	4.72
	German nurses in Germany	2.10	0.03	0.72
	Total	1.93	1.35	0.05
General motivation	Kosovar nurses in Kosovo	1.72	0.05	0.79
	Kosovar nurses in Germany	1.79	0.09	0.59
	German nurses in Germany	2.09	0.02	0.53
	Total	1.95	0.65	0.02
Thoughts about the job	Kosovar nurses in Kosovo	1.65	0.06	0.98
	Kosovar nurses in Germany	1.73	0.11	0.74
	German nurses in Germany	0.11	0.04	0.80
	Total	1.54	0.86	0.03
Quantitative demands	Kosovar nurses in Kosovo	3.23	0.06	0.91
	Kosovar nurses in Germany	2.91	0.12	0.77
	German nurses in Germany	2.76	0.04	0.86
	Total	2.92	0.90	0.03
Emotional demands	Kosovar nurses in Kosovo	2.80	0.14	2.29
	Kosovar nurses in Germany	3.33	0.13	0.88
	German nurses in Germany	2.64	0.04	0.86
	Total	2.73	1.50	0.05
Demands to hide emotions	Kosovar nurses in Kosovo	3.33	0.07	1.11
	Kosovar nurses in Germany	3.20	0.14	0.95
	German nurses in Germany	2.99	0.04	0.95
	Total	3.11	1.02	0.04
Patient-related stressors	Kosovar nurses in Kosovo	3.20	0.07	1.14
	Kosovar nurses in Germany	2.16	0.66	4.35
	German nurses in Germany	3.09	0.03	0.76
	Total	3.07	1.35	0.05
Time pressure	Kosovar nurses in Kosovo	3.29	0.05	0.87
	Kosovar nurses in Germany	3.43	0.11	0.72
	German nurses in Germany	2.64	0.04	0.84
	Total	2.90	0.91	0.03
Work–family privacy conflict	Kosovar nurses in Kosovo	2.47	0.07	1.11
	Kosovar nurses in Germany	2.82	0.15	0.99
	German nurses in Germany	2.59	0.04	0.98
	Total	2.56	1.03	0.04
General assessment of the organization	Kosovar nurses in Kosovo	2.87	0.07	1.15
	Kosovar nurses in Germany	2.09	0.31	0.12
	German nurses in Germany	2.82	0.02	0.52
	Total	2.83	0.81	0.03
Leadership quality	Kosovar nurses in Kosovo	2.50	0.07	1.12
	Kosovar nurses in Germany	2.87	0.16	1.05
	German nurses in Germany	2.79	0.04	0.92
	Total	2.69	1.00	0.04
Supervisor feedback	Kosovar nurses in Kosovo	2.49	0.07	1.26
	Kosovar nurses in Germany	3.24	0.17	1.10
	German nurses in Germany	2.76	0.05	0.08
	Total	2.70	1.16	0.04
Job satisfaction	Kosovar nurses in Kosovo	2.16	0.08	0.53
	Kosovar nurses in Germany	2.39	0.03	0.60
	German nurses in Germany	2.76	0.17	1.10
	Total	2.36	1.38	0.05

**Table 3:** Results of the Bonferroni test for the nine target factors.

Factors of Work Ability	Participant Group	Comparison Group	Bonferroni Test		
			Difference in Means	SE	*p*-Value
Physical Health	Kosovar nurses in Kosovo	Kosovar nurses in Germany	0.397	0.099	<0.001
		German nurses in Germany	0.142	0.051	0.016
	Kosovar nurses in Germany	Kosovar nurses in Kosovo	−0.397	0.099	<0.001
		German nurses in Germany	−0.255	0.096	0.023
	German nurses in Germany	Kosovar nurses in Kosovo	−0.142	0.051	0.016
		Kosovar nurses in Germany	0.255	0.096	0.023
Mental Health	Kosovar nurses in Kosovo	Kosovar nurses in Germany	0.256	0.143	0.222
		German nurses in Germany	0.386	0.074	<0.001
	Kosovar nurses in Germany	Kosovar nurses in Kosovo	−0.256	0.143	0.222
		German nurses in Germany	0.13	0.137	0.99
	German nurses in Germany	Kosovar nurses in Kosovo	−0.386	0.074	<0.001
		Kosovar nurses in Germany	−0.13	0.137	0.99
Social Health	Kosovar nurses in Kosovo	Kosovar nurses in Germany	0.281	0.105	0.023
		German nurses in Germany	0.069	0.054	0.609
	Kosovar nurses in Germany	Kosovar nurses in Kosovo	−0.281	0.105	0.023
		German nurses in Germany	−0.212	0.101	0.106
	German nurses in Germany	Kosovar nurses in Kosovo	−0.069	0.054	0.609
		Kosovar nurses in Germany	0.212	0.101	0.106
Cognitive stress symptoms	Kosovar nurses in Kosovo	Kosovar nurses in Germany	0.417	0.125	0.003
		German nurses in Germany	0.013	0.061	0.99
	Kosovar nurses in Germany	Kosovar nurses in Kosovo	−0.417	0.125	0.003
		German nurses in Germany	−0.404	0.12	0.002
	German nurses in Germany	Kosovar nurses in Kosovo	−0.013	0.061	0.99
		Kosovar nurses in Germany	0.404	0.12	0.002
Emotional demands	Kosovar nurses in Kosovo	Kosovar nurses in Germany	−0.53	0.245	0.092
		German nurses in Germany	0.158	0.113	0.485
	Kosovar nurses in Germany	Kosovar nurses in Kosovo	0.53	0.245	0.092
		German nurses in Germany	0.689	0.234	0.011
	German nurses in Germany	Kosovar nurses in Kosovo	−0.158	0.113	0.485
		Kosovar nurses in Germany	−0.689	0.234	0.011
Patient-related stressors	Kosovar nurses in Kosovo	Kosovar nurses in Germany	1.037	0.219	<0.001
		German nurses in Germany	0.113	0.101	0.792
	Kosovar nurses in Germany	Kosovar nurses in Kosovo	−1.037	0.219	<0.001
		German nurses in Germany	−0.923	0.212	< 0.001
	German nurses in Germany	Kosovar nurses in Kosovo	−0.113	0.101	0.792
		Kosovar nurses in Germany	0.923	0.212	<0.001
Time pressure	Kosovar nurses in Kosovo	Kosovar nurses in Germany	−0.143	0.139	0.919
		German nurses in Germany	0.648	0.064	<0.001
	Kosovar nurses in Germany	Kosovar nurses in Kosovo	0.143	0.139	0.919
		German nurses in Germany	0.79	0.135	<0.001
	German nurses in Germany	Kosovar nurses in Kosovo	−0.648	0.064	<0.001
		Kosovar nurses in Germany	−0.79	0.135	<0.001
General assessment of the organization	Kosovar nurses in Kosovo	Kosovar nurses in Germany	0.788	0.31	0.034
		German nurses in Germany	0.059	0.063	0.99
	Kosovar nurses in Germany	Kosovar nurses in Kosovo	−0.788	0.31	0.034
		German nurses in Germany	−0.729	0.308	0.055
	German nurses in Germany	Kosovar nurses in Kosovo	−0.059	0.063	0.99
		Kosovar nurses in Germany	0.729	0.308	0.055
Supervisor feedback	Kosovar nurses in Kosovo	Kosovar nurses in Germany	−0.75	0.192	<0.001
		German nurses in Germany	−0.267	0.087	0.007
	Kosovar nurses in Germany	Kosovar nurses in Kosovo	0.75	0.192	<0.001
		German nurses in Germany	0.483	0.186	0.029
	German nurses in Germany	Kosovar nurses in Kosovo	0.267	0.087	0.007
		Kosovar nurses in Germany	−0.483	0.186	0.029

## Data Availability

Data are contained within the article.

## Public Involvement Statement

There was no public involvement in any aspect of this research.

## Guidelines and Standards Statement

This research has been accomplished following the instructions set by the EQUATOR checklist for this quantitative research paper.

## Abbreviations

The following abbreviations are used in this manuscript:

WiN	Nurses Working Capability
WA	Work Ability
WAI	Work Ability Index

## Appendix A. Overview of Target Work Ability Factors

The individual work ability factors consist of 185 items across the following domains: Health (39 items); Training/Competence (15 items), Values/Attitude (8 items), and Work (123 items). All items are rated on a five-point Likert scale. In this study, these individual factors from the WiN Screening Manual (11) were grouped into 40 target work ability factors, consistent with the instrument. Table A1 presents the mapping of individual factors to their corresponding target work ability factors.

**Table A1:** Mapping of individual work ability factors to the target work ability factors.

Target Work Ability Factors	Instrument	Number of Items
**Health**		
The general state of health	EQ-5D; COPSOQ	1
Physical health	DigA	18
Mental health	DigA	4
Social health	DigA	6
Burnout	CBI	6
Cognitive stress symptoms	COPSOQ	4
**Training competence**		
Qualification requirements	TAA	3
Learning requirements	TAA	3
Initial qualification conditions	TAA	3
Transferability of qualifications	TAA	3
Applicability of the qualifications	TAA	3
**Values**		
Motivation to work	DigA	5
General motivation	DigA	3
**Work**		
Thoughts about the job	CBI	1
*Resources*		
Human resources	TAA	2
Spatial resources	TAA	4
Material equipment	TAA	3
Unfavorable working environment	TAA	6
Risk of injury/illness	TAA	4
*Demands*		
Cognitive demands	TAA	3
Quantitative demands	COPSOQ	4
Emotional demands	COPSOQ	3
Demands to hide emotions	COPSOQ	2
Patient-related stressors	TAA	5
Time pressure	TAA	6
Physical stress	TAA	5
Work–family privacy conflict	Work–Family Conflict Scale	5
*Organization*		
General assessment of the organization	DigA	20
Transparency of nursing tasks	TAA	3
Transparency of tasks in the hospital	TAA	4
Transparency over time	TAA	3
Scope of activity	TAA	7
Opportunities for participation	TAA	4
Organizational stressors	TAA	6
Contradictory orders	TAA	4
Work interruptions	TAA	4
Loss of quality	TAA	4
*Leadership*		
Leadership quality	COPSOQ	4
Supervisor feedback	TAA	2
*Work in general*		
Job satisfaction	COPSOQ	7

Original Source Instruments Used in the WiN Screening Manual

1. Work Ability Index (WAI), Ilmarinen et al.
2. Copenhagen Psychosocial Questionnaire (COPSOQ)—Short German Version, Kristensen et al.
3. Diagnosis of Health-Promoting Work (DigA), German Federal Institute for Occupational Safety and Health
4. Activity and Work Analysis Procedures (TAA—Tätigkeits- und Arbeitsanalyseverfahren)
5. Copenhagen Burnout Inventory (CBI), Kristensen et al.
6. EQ-5D-5L (EuroQol five-dimensions, five-level version), EuroQol Group
7. Work–Family Conflict Scale, Carlson et al.
